# Antiplatelet Activity of Tussilagone via Inhibition of the GPVI Downstream Signaling Pathway in Platelets

**DOI:** 10.3389/fmed.2020.00380

**Published:** 2020-07-29

**Authors:** Jing Zhou, Ru-Ping Yang, Wei Song, Hui-Min Xu, Yong-Hui Wang

**Affiliations:** ^1^Department of Pharmacy, Zhumadian Central Hospital, Zhumadian, China; ^2^Department of Pharmacy, Xiangyang Central Hospital, Affiliated Hospital of Hubei University of Arts and Science, Xiangyang, China; ^3^Department of Pharmacy, Renmin Hospital, Wuhan University, Wuhan, China; ^4^Department of Pharmacy, Second Affiliated Hospital, Zhejiang University School of Medicine, Hangzhou, China

**Keywords:** tussilagone, sesquiterpenoid, antiplatelet, thrombosis, glycoprotein VI pathway

## Abstract

Tussilagone is a sesquiterpenoid extracted from *Tussilago farfara* and is used as an oriental medicine for asthma and bronchitis. Although previous studies have shown that tussilagone has an inhibitory effect on platelet aggregation, no studies have been performed to investigate its precise effect on platelets, and the underlying mechanism remains unclear. In the present study, we showed that tussilagone inhibited platelet aggregation induced by collagen, thrombin and ADP, as well as platelet release induced by collagen and thrombin, in mice. Tussilagone decreased P-selectin expression and αIIbβ3 activation (JON/A binding) in activated platelets, which indicated that tussilagone inhibited platelet activation. Moreover, tussilagone suppressed platelet spreading on fibrinogen and clot retraction. The levels of phosphorylated Syk, PLCγ2, Akt, GSK3β, and MAPK (ERK1/2 and P38) and molecules associated with GPVI downstream signaling were downregulated in the presence of tussilagone. In addition, tussilagone prolonged the occlusion time in a mouse model of FeCl_3_-induced carotid artery thrombosis and had no effect on mouse tail bleeding time. These results indicate that tussilagone inhibits platelet function *in vitro* and *in vivo* and that the underlying mechanism involves the Syk/PLCγ2-PKC/MAPK and PI3K-Akt-GSK3β signaling pathways downstream of GPVI. This research suggests that tussilagone is a potential candidate antiplatelet drug for the prevention of thrombosis.

## Introduction

Platelets play important roles in hemostasis, thrombosis, inflammation, immunity, tumor metastasis and cardiovascular diseases, such as heart failure, ischemic stroke, and acute coronary syndrome (1–6). Platelets are activated by a variety of receptor-mediated stimulants, such as collagen, thrombin, ADP and thromboxane A2 (TxA2), which promotes a signaling cascade within platelets, resulting in platelet deformation, release of granular substances, and synthesis of thromboxane to promote the formation of thrombus (2). Antiplatelet therapy is still paramount to the management of these diseases.

Recent studies highlighted the importance of platelet glycoprotein (GP) VI receptor, widely known as the major receptor for collagen, and also binds to laminins, immobilized fibrinogen and fibrin (7, 8). Binding of GPVI to collagen induces tyrosine phosphorylation of the immune-receptor tyrosine-based activation motif (ITAM) in the cytoplasmic tail of the FcRγ chain, leading to a tyrosine phosphorylation-regulated cascade that involves Syk and PLCγ2 (9). Platelet GPVI represents an attractive new target because it is only expressed on platelets and megakaryocytes. In addition, GPVI blockade has been demonstrated to have efficient antithrombotic potential and show beneficial effects in other diseases (10).

At present, antiplatelet therapy mainly targets signaling pathways in platelets, such as TXA2 synthesis, adenosine diphosphate (ADP)-mediated signaling, cAMP, and integrin αIIbβ3-mediated signaling pathways (11). Although current antiplatelet agents are commonly used in clinical practice, their limitations still need to be addressed. The main severe and relatively common side effect of antiplatelet therapy is a higher risk of hemorrhage, for example, gastrointestinal bleeding and intracranial and intracerebral hemorrhage (12, 13). Another limitation affecting the efficiency of many antiplatelet drugs is genetic differences in the ability to metabolize prodrugs, such as clopidogrel, acquired anaphylaxis (heparin and aspirin) and drug resistance (aspirin) (14, 15). Thus, there is a need to develop novel platelet inhibitors with better efficacy and safety.

A multitude of traditional Chinese medicines (TCMs) are thought to promote blood circulation and remove blood stasis (16–18). Sesquiterpenoids extracted from the rhizomes of *Curcuma zedoaria* have been reported to have antiplatelet effects (19). Tussilagone, a sesquiterpene, can be extracted from the flower buds of the TCM *Tussilago farfara* L. (*T. farfara*) and used as an index of chemical extraction of *T. farfara*. Recent studies have shown that tussilagone has anti-inflammatory activity (20–22), inhibits dendritic cell function (23), suppresses the production and gene expression of MUC5AC mucin (24), and suppresses angiogenesis (25). Tussilagone has also been previously reported to have an inhibitory effect on rabbit platelet aggregation (26). However, to date, there have been no other reports on the effects of tussilagone on platelets. In this study, we investigated the effects of tussilagone on platelet function and arterial thrombosis and elucidated the possible underlying mechanism of these effects.

## Materials and Methods

### Reagents and Chemicals

**Figure 1:**
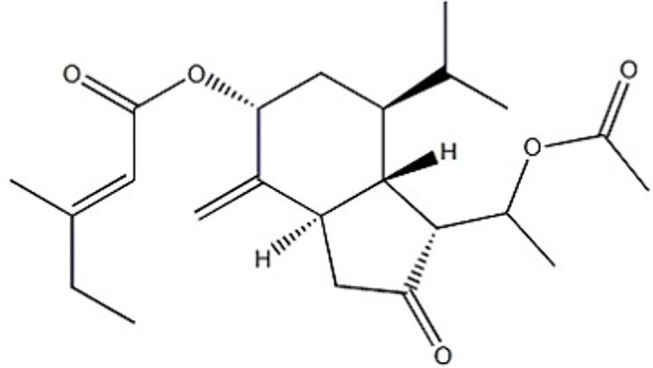
Chemical structure of tussilagone.

Tussilagone (Figure 1) was obtained from Chengdu Preferred Bio-tech Co., Ltd. (Sichuan, China). Dimethylsulfoxide (DMSO), ethylenediamine tetraacetic acid (EDTA), bovine serum albumin (BSA), adenosine diphosphate (ADP), thrombin, prostaglandin E1 (PGE_1_), and phalloidin tetramethylrhodamine isothiocyanate (phalloidin-TRITC) were purchased from Sigma Chemicals (St. Louis, MO, USA). Collagen (Type I, from equine tendons suspended in an isotonic glucose solution of pH 2.7) and luciferin-luciferase were purchased from Chrono-Log Corp. (Havertown, PA, USA). Antibodies against phospho-Akt (Ser473), phospho-GSK3β (Ser9), phospho-p38 (Thr180/Tyr182), phospho-ERK1/2 (Tyr202/204), and phospho-PLCγ_2_ (Tyr1217) were purchased from Cell Signaling (Beverly, MA, USA). Antibodies against phospho-Syk (Tyr525/526) were purchased from ABclonal Technology (Wuhan, Hubei, China). Anti-AKT, anti-GSK3β, anti-Syk, anti-p38, anti-ERK1/2, anti-PLCγ_2_, anti-GAPDH and HRP-conjugated goat anti–rabbit IgG antibodies were obtained from Santa Cruz Biotechnology (Santa Cruz, CA, USA). A FITC-conjugated anti-CD62P antibody was obtained from BD Biosciences (San Jose, CA, USA). A PE-conjugated anti-αIIbβ3 antibody (JON/A) (M023-2) was obtained from Emfret Analytics (Eibelstadt, Germany). Protease and phosphatase inhibitor cocktails were purchased from EMD Millipore Chemicals (Billerica, MA, USA). The ECL Western blotting detection reagent was purchased from Pierce Chemical Co. (Rockford, IL, USA).

### Animals

Male ICR mice (weight, 18–25 g; age, 8 weeks) purchased from Hubei Experimental Animal Research Center (Wuhan, Hubei, China) were housed in a specific pathogen-free environment under standard laboratory conditions: relative humidity of 55–65%, temperature of 22–24°C, 12-h dark/light cycle (lights on at 7:00; lights off at 19:00). The mice were provided free access to food and water and acclimated for at least 1 week before the experiment. All experimental protocols and animal care procedures were carried out according to the relevant guidelines and were approved by the Ethics Committee for Experimental Animals at Zhumadian Central Hospital.

To investigate the effect of tussilagone on platelets *in vivo*, mice were separated into two groups (*n* = 8 per group). The tussilagone mice were orally administered tussilagone (10 mg/kg) daily (25), while the control mice received the same dose of corn oil. After 7 days, the mice were used for ferric chloride-induced carotid injury experiments, a tail bleeding time assay and an *ex vivo* platelet aggregation test.

### Mouse Platelet Isolation and Preparation

Washed mouse platelets and platelet-rich plasma (PRP) were prepared as described previously (27). Briefly, after mice were anesthetized with urethane by intraperitoneal injection, whole blood was drawn from the inferior vena cava into a syringe containing 3.8% sodium citrate (9:1, v/v). PRP was obtained by removing red blood cells after centrifuging the whole blood for 15 min at 150 × g. By centrifuging blood samples that contained almost no platelets at 800 × g for 15 min, the supernatant was obtained as the platelet-poor plasma (PPP) fraction. The PPP was used as a reference solution and diluent in the aggregation assay. Blood (2–3 mL) was diluted with Tyrode's buffer (137 mM NaCl, 13.8 mM NaHCO_3_, 5.5 mM glucose, 2.5 mM KCl, 20 mM HEPES, and 0.36 mM NaH_2_PO_4_, pH 7.4) containing 1 μM PGE1 and centrifuged at 160 × g for 15 min at room temperature to obtain platelets. The platelets were washed twice softly with Tyrode's buffer containing 1 μM PGE1 and 1 mM EDTA. The pelleted platelets were resuspended in Tyrode's buffer (3.0 × 10^8^/mL) and incubated for 30 min at 37°C prior to stimulation.

### Platelet Aggregation and ATP Release Assays

Platelet aggregation and ATP release assays were performed as described previously (28) by using an aggregometer (Chrono-Log Corp., Havertown, PA, USA). Briefly, washed platelet suspensions or PRP (3 × 10^8^/mL) was placed *in silicon*ized glass cuvettes at 37°C with a stir speed of 1,000 rev/min in the aggregometer. Before stimulation with different agonists, the washed platelets and PRP were preincubated with 0.4% DMSO (vehicle) or different concentrations of tussilagone (10, 20, or 40 μM) for 5 min at 37°C. CaCl_2_ (final concentration of 1 mM) was added prior to agonist stimulation. When the platelets were stimulated with agonist, platelet ATP release was measured using luciferin-luciferase reagent. The aggregation and ATP release results were recorded by Aggregolink software. The data are presented as actual traces, and the aggregation and ATP release of untreated platelets (vehicle) were set as 100%. Aggregation was assessed turbidimetrically and expressed as the percent change in light transmission.

### Clot Retraction

A total of 500 μL of washed platelet suspension (3.0 × 10^8^/mL) containing 400 μg/mL fibrinogen and 1 mM CaCl_2_ was stimulated with 5 μL of thrombin (20 U/mL) to initiate clot retraction at 37°C. Clot retraction was monitored by taking photographs at the indicated time points using a digital camera (Nikon D90, Japan).

### Platelet Spreading

Washed platelets (3.0 × 10^7^/mL) were incubated with different concentrations of Tussilagone (10, 20, or 40 μM) or vehicle (0.4% DMSO) for 5 min and permitted to spread on coverglasses coated with fibrinogen (25 μg/mL) or BSA for 45 min at 37°C in 24-well plates. Then, PBS was used to wash the coverglasses twice after the platelet suspension was removed. The adherent platelets were fixed with 2% paraformaldehyde, permeabilized with 0.1% Triton, and stained with fluorescein-labeled phalloidin. A fluorescence microscope (Nikon TE-2000S, Japan) was used to obtain images, and ImageJ software (NIH, USA) was used to calculate the platelet spreading area.

### Flow Cytometry Analysis

A FITC-conjugated anti-CD62P antibody or PE-labeled anti-αIIbβ3 antibody (JON/A) was added to washed platelets (5.0 × 10^7^/mL) pretreated with different concentrations of Tussilagone (10, 20, or 40 μM) or vehicle (0.4% DMSO) for 5 min at 37°C, and the platelets were incubated for 15 min at room temperature in the dark. Then, the samples were analyzed by flow cytometry (BD Biosciences, CA).

### Western Blotting

Resting washed platelets (3.0 × 10^8^/mL) or platelets stimulated with the agonist after being preincubated with tussilagone or vehicle for 5 min were lysed with the same volume of 2× lysis buffer (300 mM NaCl, 20 mM EGTA, 2% Triton X-100, 30 mM HEPES, 0.2 mM MgCl_2_, 2× protease inhibitor cocktail and 2× phosphatase inhibitor cocktail). The protein concentration was quantified by a BCA assay. We subjected the samples to electrophoresis on a 10% SDS polyacrylamide gel (SDS-PAGE) and transferred them to polyvinylidene difuoride (PVDF) membranes. The membranes were blocked with 5% skim milk for 1 h at room temperature before incubated with the corresponding antibodies. Then, the membranes were incubated with appropriate HRP-conjugated secondary antibodies for 1 h at room temperature. The blots were developed using a chemical luminescence imaging system (DNR Bio-imaging Systems) and analyzed by ImageJ.

### Ferric Chloride-Induced (FeCl_3_) Carotid Injury

A carotid thrombosis model was established as previously described (28). After anesthetization by intraperitoneal injection of urethane, the left common carotid artery was exposed. Blood flow was continuously monitored using a perivascular flow probe (Transonic, UK) connected to a TS420 flow meter (Transonic, UK). A 2-mm strip of filter paper saturated with 12% FeCl_3_ was applied to the carotid artery surface for 3 min. Blood flow was monitored for 30 min or until the absence of blood for 1 min after removal of the filter paper. Occlusion was defined as the time required for > 90% loss of the initial blood volume.

### Tail Bleeding Assay

The mice were anesthetized, and a 3-mm segment of the tail tip was amputated with a scalpel. Tail bleeding was monitored by gently absorbing the blood with filter paper at 30-s intervals without touching the wound site. The bleeding time was defined as the time required for bleeding to stop for more than 1 min or 30 min.

### Statistical Analysis

We analyzed the data using GraphPad Software 6.0 (Graph Pad, USA). All data were normally distributed and expressed as the means ± standard errors of the mean (SEMs). Data were analyzed by Two-tailed unpaired Student's *t*-test or one-way ANOVA followed by Tukey's multiple comparison test. *P* < 0.05 were considered statistically significant.

## Results

### Tussilagone Inhibits Platelet Aggregation

**Figure 2:**
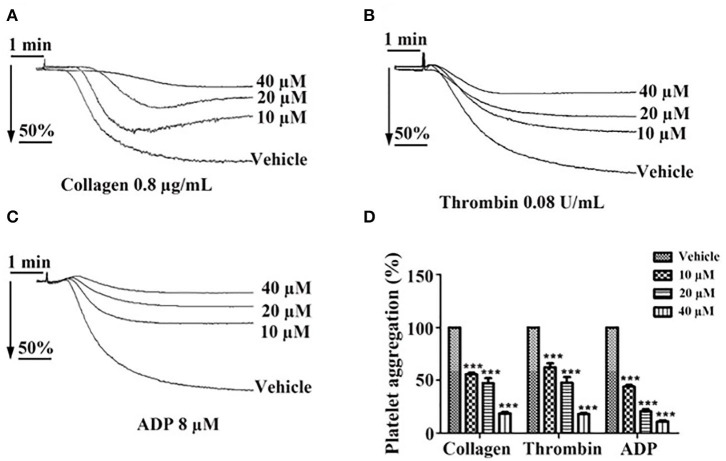
Inhibitory effect of tussilagone on platelet aggregation induced by different agonists. Preincubated with different concentrations of tussilagone (10, 20, or 40 μM) or vehicle for 5 min, platelet aggregation of washed platelets induced by **(A)** collagen (0.8 μg/mL) or **(B)** thrombin (0.08 U/mL) and platelet aggregation of PRP (3.0 × 10^8^/mL) induced by **(C)** ADP (8 μM). **(D)** The aggregation (%) were quantified. One-way ANOVA was used for analysis. The data are presented as the mean ± SEM (*n* = 4). ***p < 0.001, vs. vehicle.

Incubation of washed platelets or PRP with tussilagone (10, 20, or 40 μM) for 5 min led to a concentration-dependent inhibitory effect on platelet aggregation induced by collagen (0.8 μg/mL) (55.7 ± 1.5, 47.3 ± 4.7, and 18.7 ± 1.3%, respectively), thrombin (0.08 U/mL) (62.3 ± 3.8, 47.7 ± 5.5, and 18.0 ± 1.2%, respectively) and ADP (8 μM) (44.0 ± 1.5, 20.7 ± 2.2, and 11.0 ± 1.2%, respectively) (Figure 2).

### Tussilagone Inhibits Platelet Granule Secretion and Platelet Activation

**Figure 3:**
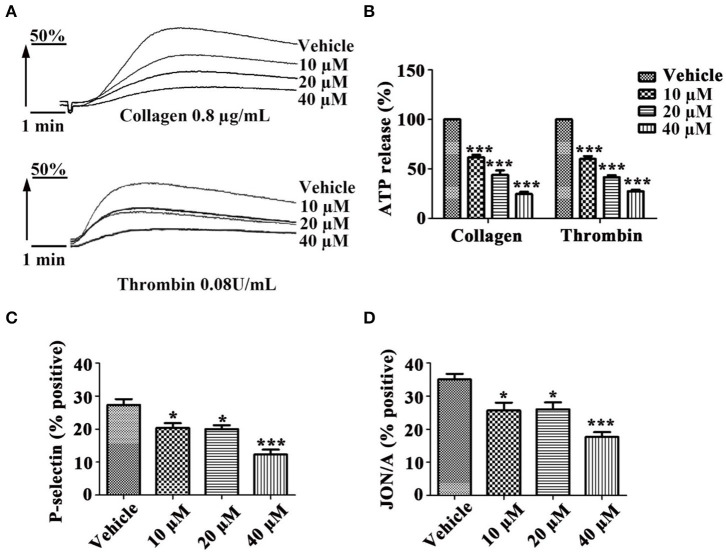
Tussilagone inhibits platelet ATP release, P-selectin expression and αIIbβ3 activation. **(A)** Representative traces of platelet ATP release induced by collagen (0.8 μg/mL) or thrombin (0.08 U/mL) were recorded. **(B)** Quantification of ATP release. **(C)** Platelet P-selectin expression and **(D)** αIIbβ3 activation (JON/A binding) were detected by flow cytometry after platelet activation. Washed platelets (5.0 × 10^7^/mL) were preincubated with different concentrations of tussilagone (10 μM, 20 μM, or 40 μM) or vehicle for 5 min at 37°C and stimulated with thrombin (0.2 U/mL) in the presence of a FITC-conjugated anti-CD62P antibody or a PE-conjugated JON/A antibody. The data are presented as the mean ± SEM (*n* = 4). One-way ANOVA was used for data analysis. *p < 0.05 and ***p < 0.001 vs. vehicle.

To investigate whether tussilagone has an effect on platelet granule secretion, washed platelets were preincubated with different concentrations of tussilagone and then stimulated with collagen (0.8 μg/mL) or thrombin (0.08 U/mL). The results showed that tussilagone (10, 20, or 40 μM) had a significant inhibitory effect on platelet ATP release induced by collagen (61.2 ± 2.3, 44.0 ± 4.5, and 24.7 ± 2.3%, respectively) and thrombin (60.0 ± 2.9, 41.7 ± 2.0, and 27.3 ± 1.8%, respectively) (Figures 3A,B). Then, we examined the effect of tussilagone on platelet activation. Washed platelets were preincubated with tussilagone before stimulation with thrombin (0.2 U/mL) and P-selectin expression and αIIbβ3 activation (JON/A binding) were detected by flow cytometry. We found that tussilagone also inhibited platelet P-selectin expression and JON/A binding (Figures 3C,D).

### Tussilagone Suppresses Platelet Spreading on Fibrinogen and Clot Retraction

**Figure 4:**
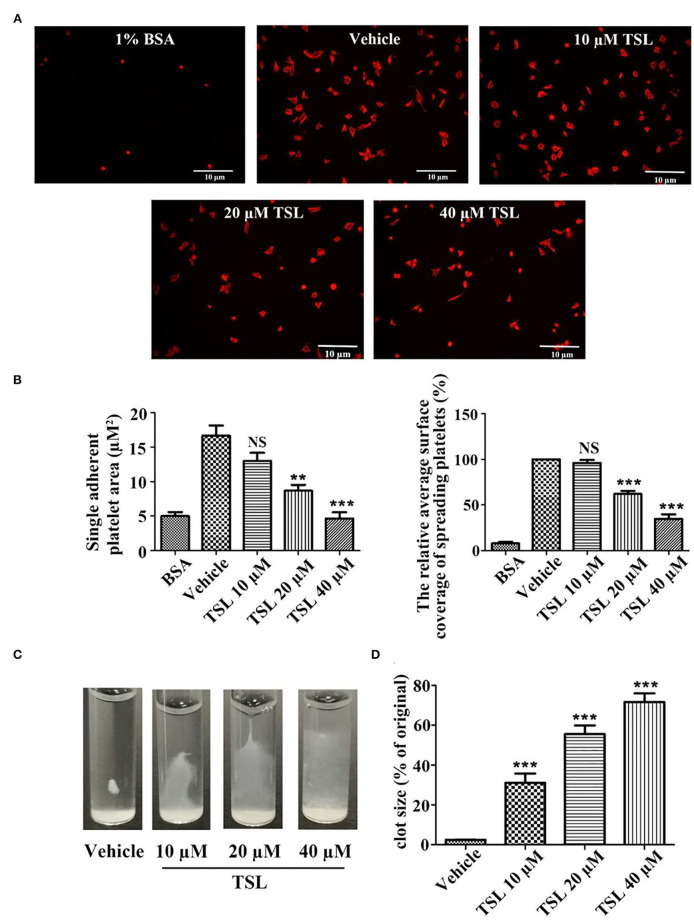
Tussilagone suppresses platelet spreading on fibrinogen-coated surfaces and clot retraction. **(A)** Representative images of platelet spreading on BSA- or fibrinogen-coated surfaces. Washed platelets (2.0 × 10^7^/mL) were preincubated with vehicle or different concentrations of tussilagone (10, 20, or 40 μM) for 5 min at 37°C before being allowed to spread on coated glass. **(B)** The area covered by single adherent platelets (left) and the relative average coverage area of spreading platelets (the values were normalized to vehicle, which was set as 100%) (right) were quantified. **(C)** Representative images of clot retraction were taken 15 min later. **(D)** Summary data of clot retraction were presented. The data are presented as the mean ± SEM (*n* = 4). One-way ANOVA was used for data analysis. **p < 0.01, ***p < 0.001, NS, not significant, vs. vehicle. TSL, tussilagone.

Platelet spreading on fibrinogen was investigated after preincubation with tussilagone (10, 20, or 40 μM). Compared to that in the vehicle group, the formation of filopodia and lamellipodia by platelets, as well as the area of single adherent platelets and relative surface coverage area were reduced in the tussilagone-treated group in a dose-dependent manner (Figures 4A,B). In addition, in the presence of tussilagone, especially at the high dose, clot retraction was also significantly suppressed (Figures 4C,D).

### Tussilagone Negatively Affects Collagen-Induced Intracellular Signaling in Platelets

**Figure 5:**
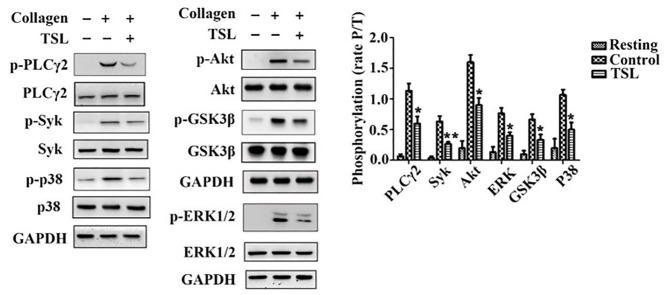
Tussilagone inhibits collagen-induced intracellular signaling in platelets. Washed platelets (3.0 × 10^8^/mL) were preincubated with vehicle or tussilagone (40 μM) for 5 min at 37°C before being stimulated with collagen (0.8 μg/mL) with stirring at 1,000 rpm in an aggregometer at 37°C and then lysed to analyze signaling molecules by Western blotting. Representative blots are shown, and the levels of PLCγ2, Syk, Akt, GSK3β, p38, and ERK1/2 phosphorylation were determined. The data are presented as the mean ± SEM (*n* = 4). One-way ANOVA was used for data analysis. *p < 0.05 and **p < 0.01 vs. vehicle. TSL, tussilagone.

To investigate how tussilagone inhibits platelet function, we examined signaling molecules in platelets after collagen stimulation. Western blotting results showed that the levels of Syk, PLCγ2, Akt, and GSK3β, as well as the phosphorylation of mitogen-activated protein kinase (MAPK) family members (ERK1/2 and P38), were decreased by tussilagone (40 μM) (Figure 5). These findings indicate that tussilagone negatively regulates GPVI signaling in platelets.

### Tussilagone Inhibits Platelet Function and Thrombosis *in vivo*

**Figure 6:**
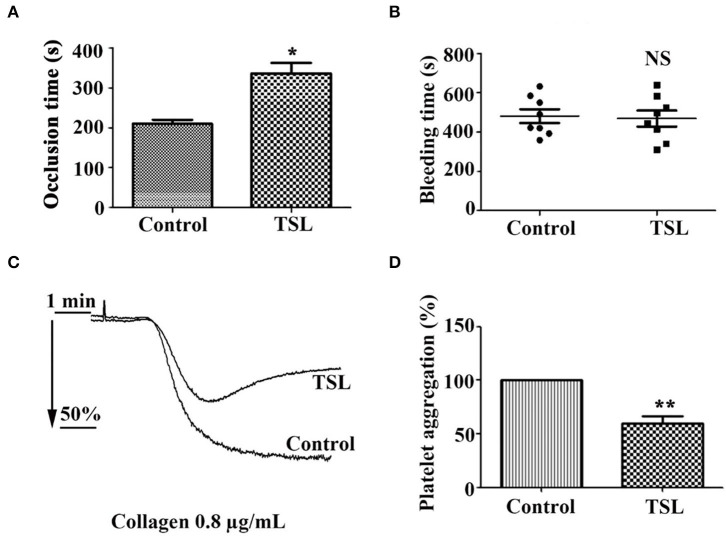
Tussilagone inhibits thrombosis *in vivo*. **(A)** Vascular occlusion times were compared between vehicle- and tussilagone-treated mice after FeCl_3_-induced injury of the carotid artery. The occlusion time represents the amount of time required to form an occlusive thrombus as described in the Materials and Methods. **(B)** The effect of tussilagone on bleeding time. The bleeding time from tail transection to complete cessation of bleeding was recorded. There were eight mice per group for **(A,B)**. **(C)** Representative traces of platelet aggregation induced by collagen (0.8 μg/mL) in control mice and tussilagone-treated mice. **(D)** The aggregation (%) was quantified, and the aggregation of control mice was taken as 100% (*n* = 4). The data are presented as the mean ± SEM. Two-tailed unpaired Student's *t*-test was used for data analysis. *p < 0.05, **p < 0.01, NS, not significant, vs. control. TSL, tussilagone.

To determine the inhibitory effect of tussilagone on platelets *in vivo*, mice were given tussilagone orally for 7 days, while the control mice were given corn oil. The occlusion time of the carotid artery and tail bleeding time were measured. Compared with control FeCl_3_-induced carotid injury model mice, FeCl_3_-induced carotid injury model mice treated with tussilagone (10 mg/kg) exhibited prolonged thrombotic occlusion time (Figure 6A). The tail bleeding time assay showed that compared to control, tussilagone had no effect on tail bleeding time (Figure 6B). In addition, compared to those from control mice, washed platelets from tussilagone-treated mice showed decreased aggregation induced by collagen (Figures 6C,D). These results indicate that tussilagone inhibits platelet function *in vivo* without affecting bleeding time.

## Discussion

Tussilagone is a component extracted from the flower buds of *Tussilago farfara* L. (Kuan dong hua) and is used as a traditional oriental medicine for asthma and bronchitis. Tussilagone was shown to have an effect on hemodynamics and platelet aggregation as early as 1987 (26, 29). However, to date, no further studies on the effects of tussilagone on platelets have been conducted. In the present study, we found that tussilagone at concentrations of 10, 20, and 40 μM inhibited platelet aggregation induced by collagen, thrombin and ADP; platelet ATP release induced by collagen, thrombin; P-selectin expression and αIIbβ3 activation in activated platelets; platelet spreading; and clot retraction. In addition, tussilagone reduced the formation of thrombus in a carotid artery model induced by FeCl_3_ and did not affect tail bleeding time. Mechanistically, tussilagone decreased the phosphorylation of molecules involved in the Syk/PLCγ2-PKC/MAPK and PI3K-Akt-GSK3β signaling pathways downstream of GPVI.

Platelet aggregation contributes to thrombosis and growth. When the endothelial cell monolayer is breached, collagen fibrils within the vessel wall become exposed to the circulation to form a complex with von Willebrand factor (vWF). Platelets are captured when the glycoprotein (GP) Ibα on the platelet surface binds to vWF, which is essential for platelet activation (30). Platelet aggregate formation is dependent on the local generation or release of soluble agonists, such as ADP, α-thrombin and thromboxane A2 (TXA2), which are of particular importance in the process that converts αIIbβ3 into a high-affinity receptor for soluble adhesive proteins (31). Tussilagone had a strong inhibitory effect on collagen-, thrombin- and ADP-induced platelet aggregation in a concentration-dependent manner (Figure 1). These results led us to further investigate the effect of tussilagone on platelets.

Molecules stored in specific granules are released when platelets are activated, and this is an important step for hemostasis, thrombosis, and other pathophysiological processes (32). There are three main granules: α-granules, dense granules and lysosomes contained in platelets (33). α-Granules are unique to platelets, and they are the most abundant platelet granules and contain many proteins, such as P-selectin, fibrinogen, and vWF. Dense granules are rich in ADP, ATP and 5-HT (34). When platelet activation occurs, ATP is released from dense granules, intracellular P-selectin is released from α-granules and exposed to the platelet membrane and conformational changes in integrin αIIbβ3 occur (35). Tussilagone not only inhibited platelet ATP release induced by collagen and thrombin (Figures 3A,B) but also suppressed platelet P-selectin expression and αIIbβ3 activation (JON/A binding) stimulated by thrombin (Figures 3C,D). These results suggest that tussilagone has an inhibitory effect on platelet activation.

Integrin αIIbβ3, as a platelet-abundant specific adhesive receptor, initiates outside-in signaling and plays a complex pivotal role in platelet physiology, such as spreading, adhesion, aggregation, clot formation and clot retraction (36). All these processes accelerate stable thrombus formation (37). Tussilagone also had an inhibitory effect on platelet spreading (Figures 4A,B) and clot retraction (Figures 4C,D). This indicates that tussilagone can inhibit outside-in signaling in platelets.

To study the underlying molecular mechanism of the antiplatelet function of tussilagone, we focused on GPVI signaling stimulated by collagen. Collagen binds to GPVI, leading to the Src kinase-dependent phosphorylation of the two conserved tyrosines, the binding of the tandem SH2 domains of Syk and the initiation of a signaling cascade that activates PLCγ2. Activated PLCγ2 induces the formation of the second messengers inositol 1,4,5-trisphosphate (IP3) and 1,2-diacylglycerol (DAG), resulting in the mobilization of intracellular Ca^2+^ stores and the activation of PKC/MAPK (ERK1/2, P38, JNK), respectively (38). Furthermore, MAPK ERK1/2, p38, and JNK1 in platelets are also activated by other stimuli, such as thrombin, vWF and ADP, and contribute to hemostasis and thrombosis (39). In addition, the stimulation of the PI3K/Akt pathway by GPVI-mediated platelet activation and integrin engagement play important roles in thrombus formation and stabilization (40). Moreover, GSK3β has been found in platelets and acts as an Akt effector to regulate platelet activation (41). The phosphorylation of GSK3β by Akt is related to decreased GSK3β activity, which leads to negative regulation of platelet function and thrombosis (42). Our data showed that tussilagone decreased the phosphorylation of Syk, PLCγ2, MAPK (P38 and ERK1/2), Akt and its substrate GSK3β downstream of collagen-induced GPVI signaling (Figure 5).

To further explore the inhibitory effect of tussilagone on platelets *in vivo*, mice were orally administered tussilagone for 7 days to evaluate acute thrombus formation following FeCl_3_-induced injury of the carotid artery. Our study showed that tussilagone markedly prolonged the occlusion time (Figure 6A). In addition, collagen-induced platelet aggregation was reduced in tussilagone-treated mice compared to control mice (Figures 6C,D), which was consistent with the inhibitory effect of tussilagone on platelet aggregation *in vitro*. In view of the multitarget and multilevel characteristics of traditional Chinese medicines, antiplatelet effects have been widely explored (43). However, certain ingredients with antiplatelet effects, such as atractylodes lactone compounds (44) and miltirone (28), have been reported to cause side effects associated with bleeding. Therefore, it is important to find safe and effective antiplatelet agents. Fortunately, we found that tussilagone had no effect on tail bleeding time in mice (Figure 6B). These results indicate that tussilagone inhibits platelet function *in vivo* without affecting bleeding.

In conclusion, our results showed that tussilagone inhibited platelet aggregation, granule release, αIIbβ3 activation, platelet spreading on fibrinogen and clot retraction. In addition, tussilagone also inhibited thrombosis in a FeCl_3_-induced carotid artery model and had no effect on bleeding time. These results illustrate that tussilagone has a potent inhibitory effect on platelet function *in vitro* and *in vivo*. The underlying mechanism is possibly the inhibition of the Syk-PLCγ2-PKC/MAPK and PI3K-Akt-GSK3β pathways downstream of GPVI signaling cascades. The research suggests that tussilagone has the potential to be developed as a new antiplatelet candidate for the prevention of thrombotic disorders.

## Data Availability Statement

The raw data supporting the conclusions of this article will be made available by the authors, without undue reservation, to any qualified researcher.

## Ethics Statement

The animal study was reviewed and approved by Ethics Committee for Experimental Animals of Zhumadian Central Hospital.

## Author Contributions

WS and R-PY contributed to the conception, design of the study, and critically revised the article. JZ, H-MX, and Y-HW acquired the data. Y-HW performed the statistical analysis. JZ and H-MX wrote the first draft of the manuscript. All authors contributed to manuscript revision and read and approved the submitted version.

## Conflict of Interest

The authors declare that the research was conducted in the absence of any commercial or financial relationships that could be construed as a potential conflict of interest.
